# Study of the biological characteristics of human umbilical cord mesenchymal stem cells after long-time cryopreservation

**DOI:** 10.1007/s10561-021-09973-1

**Published:** 2022-01-23

**Authors:** Mingqi Zhang, Yan Zhao, Le Wang, Yuqiang Zheng, Hui Yu, Xiaoming Dong, Wei He, Zhengqin Yin, Zhuoshi Wang

**Affiliations:** 1 Department of Stem Cell Center of Precision Medicine Innovation Institute, He University, Hunnan District, No.66 Sishui Street, Shenyang, 110163 China; 2 Liaoning Key Lab of Ophthalmic Stem Cells, He University, Shenyang, China; 3 Liaoning Province Ophthalmic Stem Cell Clinical Application Research Center, He Eye Hospital, Shenyang, China

**Keywords:** Human umbilical cord mesenchymal stem cells, Cryopreservation, Cell proliferation, Surface marker, Multilineage differentiation, Karyotype

## Abstract

**Abstract:**

Human umbilical cord mesenchymal stem cells (hUC-MSCs) have considerable potential in cell therapy. Cryopreservation represents the gold standard in cell storage, but its effect on hUC-MSCs is still not well understood. The aim of this study was to investigate the effect of one year of cryopreservation and thawing on the biological characteristics of hUC-MSCs from the same donors. Fresh hUC-MSCs were cryopreserved in commercial freezing medium (serum-free CellBanker 2) at passage 2. After one year of cryopreservation, the hUC-MSCs were thawed and subcultured to passage 4. The comparison was performed in terms of followings: cell count, viability, morphology, proliferation capacity, differentiation potential and chromosomal stability. The total cell count and viability of hUC-MSCs before and after one year of cryopreservation were 1 × 10^7^ and 96.34% and 0.943 × 10^7^ and 93.81%, respectively. Cryopreserved and fresh hUC-MSCs displayed a similar cell doubling times, expressed the markers CD73, CD90, CD105 and were negative for the markers CD34, CD45, and HLA-DR. Karyotypes were found to be normal after one year of cryopreservation. The trilineage differentiation properties were maintained after cryopreservation. However, when compared to freshly isolated hUC-MSCs from the same donor, cryopreserved hUC-MSCs exhibited decreased expression of osteogenesis- and chondrogenesis-related genes including Runx2, Sox9, and Col1a1, and increased expression of adipogenesis-related genes. These results demonstrated that cryopreservation did not affect cell morphology, surface marker expression, cell viability, proliferative capacity, or chromosomal stability. However, the osteogenic and chondrogenic differentiation capacities of cryopreserved hUC-MSCs were slightly reduced compared with those of fresh cells from the same donor.

**Graphical abstract:**

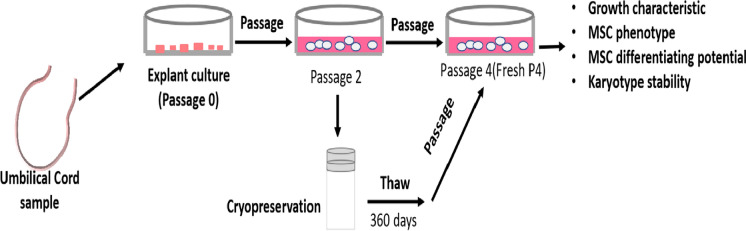

## Introduction

In recent studies in the field of regenerative medicine, human mesenchymal stem cells (hMSCs) have shown great potential for use in the treatment of various diseases (Li et al. 2015; Yang et al. 2020). MSCs can be administered as “off-the-shelf” cell products for immunotherapies and regenerative medicine (Guan et al. 2014; Zhao et al. 2019; Pham and Vu 2020). Currently, there are 1261 clinical trials studying MSCs that are registered with FDA, of which 82 trials have advanced to phase 3 and phage 4 (Search for “mesenchymal stem cells” at www.clinicaltrials.gov).

MSCs can be harvested from multiple tissues and organs, but over 80% of clinical trials use MSCs derived from bone marrow (BM), adipose tissue (AT), and umbilical cords (UCs) (Berebichez-Fridman and Montero-Olvera 2018). Human umbilical cord-derived MSCs (hUC-MSCs) can be obtained without causing pain and exhibit faster self-renewal. hUC-MSCs have shown very significant anti-inflammatory, immunomodulatory and tissue repair effects with low immunogenicity, strong migratory abilities, and neurotropic properties, which makes them ideal candidates for allogeneic cell-based therapies to treat several diseases (Nagamura-Inoue and He 2014; Watson et al. 2015). Based on the remarkable biological characteristics of hUC-MSCs, they have been used in the treatment of retinal diseases, for which there is currently no effective therapeutic strategy (Adak et al. 2021). In a phase I/II clinical trial involving 32 patients with advanced retinal pigmetosa (RP), Yin et al. tested the safety and efficacy of the intravenous adiministration of 1 × 10^8^ UC-MSCs to advanced RP patients, and they found that 81% of the visual function of the patients was maintained for 12 months (Zhao et al. 2020). Intravenous infusion of hUC-MSCs has been used in the treatment of different systemic diseases, and different degrees of improvement have been achieved (Gu et al. 2018).

MSCs are currently classified as cell-based medicinal products, and MSCs have to be expanded on a large-scale to meet the needs of clinical trials. In most clinical trials, recipients are intravascularly administered 1–5 × 10^6^/kg MSCs (Juliana et al. 2019). Each type of stem cells is prepared by a different procedure and different types of stem cells are known to differ in terms of yield and quality. Therefore, there is an urgent clinical need to establish universally accepted materials and operating procedures for the safe, reproducible, and large-scale expansion of MSCs.

Fetal bovine serum (FBS) has traditionally been used as a supplement for culturing stem cells. However, FBS poses several safety concerns, including significant batches variation among batches, contamination with animal pathogens and xenoimmunization (Yong et al. 2015). To make large amounts of clinical-grade hUC-MSCs for therapy, there is a need to develop defined xeno-free large-scale production systems. Recently, human platelet lysate (hPL) has attracted substantial attention as a substitute for fetal bovine serum (FBS) for clinical-grade cell expansion. Previous studies have shown that adipose-derived stem cells and bone morrow-derived stem cells exhibited a significantly faster growth in hPL-supplemented culture medium than cultured in FBS-supplemented culture medium (Romaldini et al. 2018; Fekete et al. 2012). In this study, we used xeno-free hPL-supplemented medium to expand of hUC-MSCs.

Another important element for the commercial and clinical application of MSCs is cryopreservation and long-term storage. Traditionally, the cryopreservation procedure has been carried out using DMSO cryoprotectant solution with gradient cooling to −80°C, and then, cells were stored in liquid nitrogen. Previous studies indicated that cryostorage exposed cells to physical insult causing cell intracellular structural and molecular changes that compromised clinical benefits (Mullers et al. 2019). Thus, verifying the characteristics of cryopreserved hUC-MSCs is highly important for their clinical applications. Previous studies about the effects of cryopreservation on hUC-MSCs are difficult to interpret, as limited data on the long-term cryopreservation of MSCs are available. Thus, investigations into the properties of cryopreserved MSCs may be informative.

In the present study, we compared freshly isolated hUC-MSCs with hUC-MSCs cryopreserved for 12 months in terms of their cell viability, surface marker expression, multilineage differentiation potential, and karyotype analysis. The study will provide the experimental evidence for further application in the regenerative medicine field.

## Materials and methods

### hUC-MSCs isolation and culture

This study was approved by the Medical Ethics Committee of He University, and informed patient consent was obtained from all the subjects (NO.IRB (2019) K007.01). Newborn umbilical cord tissues were obtained from 3 different donors after full-term births (maternal age range of 32–38 years old, all mothers of twins) and transported to the stem cell laboratory for stem cell isolation. The mother’s medical history was screened, and a blood sample was tested for specific human pathogens, such as human immunodeficiency virus 1/2 (HIV1/2), hepatitis B and C virus (HBV and HCV), and syphilis. The umbilical cord tissues were sprayed with 75% ethanol and transferred to saline solution (0.9% NaCl). The umbilical cord was cut into 2-cm sections, and the umbilical veins, arteries, and adventitia were removed. Finally, Wharton’s jelly tissue was obtained. The tissues were transferred to petri dishes containing α-MEM (Dakewei, China) supplemented with 5% human platelet lysate (UltraGRO, Helios Bioscience). The dishes were placed in a 5% CO_2_ incubator at 37°C for 8–12 days. Once the cells had reached confluence, the adherent cells (passage 0) were detached with 0.125% trypsin and expanded in multilayer flasks. At passage 2, the cells were detachment from the flasks, and some of the cells were subsequently expanded, while the rest of the cells were frozen for future expansions. Cells in passage 4 were required for all the assays.

### Cryopreservation

Approximately 1 × 10^7^ cells at passage 2 were transferred to 2 ml cryovial with the commercial serum-free cryoprotective agent CellBanker 2 (ZENOAQ, Japan). All the cryovials were placed in isopropanol-buffered freezing containers for controlled freezing at a rate of −1°C/minute to −80°C. The cells were stored at -80°C overnight and then transferred to liquid nitrogen (−196°C) the next day. After 12 months of cryopreservation, the cells from three donors were rapidly thawed in a 37°C water bath, subcultured to passage 4 under the same conditions as those described above and used to evaluate cell phenotype, viability, and functional properties. Freshly prepared human mesenchymal stem cells (abbreviated as “F-hUC-MSCs”) and human mesenchymal stem cells cryopreserved for 12 months (abbreviated as “C-hUC-MSCs”) were used in this study.

### Microbiological testing

The cells were tested for the presence of aerobic and anaerobic bacteria (BactALERT), mycoplasma by PCR assay and endotoxin detection by Gel Clot LAL Assay. The cell identity was validated by short tandem repeat (STR) profiling (Liaoning Biotechnology, China).

### Cell viability and proliferation potential

The total cell count and viability in the fresh hUC-MSC and cryopreserved hUC-MSC groups were measured by trypan blue dye exclusion assay. Ten microliters of cell suspension was mixed with 10 μL 0.4% w/v trypan blue solution for 5 min, and the dead cells were stained and counted with a countess automated cell counter (Countstar, IC1000). The proliferation potential of hUC-MSCs from the two groups was assessed by estimating the population doubling time (PDT). The PDT was calculated with the following formula: PDT = log_2_ (N_X_/N_0_) × T. The total number of cells initially attached to the culture flask was represented by N_0_, the cell count after detachment was reprensented by N_x,_ and the culture duration in hours was represented by T (Davidson et al.^2015^).

### Cell phenotype

Both fresh and thawed hUC-MSCs at passage 4 were used to determine the expression level of surface markers by flow cytometry. To perform phenotype characterization, the cells were incubated with antibodies labeled with fluorochromes as follows: CD90-PE, CD73-PE, CD105-PE, CD34-FITC, CD45-FITC, and HLA-DR-FITC (all antibodies purchased from BD Biosciences). The specific method was as follows: each primary antibody was added per 1 × 10^6^ cells separately and incubated at room temperature for 1 h. Isotype-matched antibodies were used as controls. Then, the cells were washed again with PBS and analyzed by flow cytometry (FACS Calibur C6, BD).

### Multilineage differentiation potential assays

For adipogenic differentiation, hUC-MSCs were seeded at a density of 2 × 10^4^ cells/cm^2^ and were cultured in adipogenic induction medium (OriCell™, Cyagen, China) for 21 days. After 21 days, the differentiation of the hUC-MSCs into adipogenic-like cells was indicated by the appearance of lipid droplets stained by Oil Red O (Cyagen, China). Furthermore, the adipogenic potential of hUC-MSCs before and after 360 days of cryopreservation was confirmed by determining the gene expression levels of adipogenic markers using real-time polymerase chain reaction (real-time PCR). Peroxisome proliferator-activated receptor-γ (PPARγ) and adiponectin served as specific markers of adipogenic differentiation.

For osteogenic differentiation, hUC-MSCs were seeded in 6-well plates precoated with 0.1% gelatin. Subsequently, the medium was substituted with osteogenic differentiation medium (OriCell™, Cyagen, China), and the cells were cultured until they reached 60–70% confluence. After 2–4 weeks of induction, the differentiation of the hUC-MSCs into osteogenic-like cells was indicated by calcium deposition, which was stained using Alizarin Red (Cyagen, China). Furthermore, the osteogenic potential of hUC-MSCs before and after 360 days of cryopreservation was confirmed by determining the gene expression levels of osteogenic markers. Runt-related transcription factor 2 (Runx2) and osteopontin (OPN) served as markers of osteogenic differentiation.

For chondrogenic differentiation, 5 × 10^5^ hUC-MSCs were pelleted in 15-mL tubes and cultured with the chondrogenic differentiation medium (OriCell™, Cyagen, China) for 21 days, and the medium was refreshed every 3 days. The chondroid pellets were fixed in 4% neutral buffered formalin and processed according to the standard histological procedures to generate tissue sections. Finally, the tissue sections were stained with Alcian Blue (Cyagen, China). Furthermore, the chondrogenic potential of hUC-MSCs before and after 360 days of cryopreservation was confirmed by determining the gene expression levels of the chondrogenic marker Sry-related HMG box-9 (Sox9) and Col1a1 served as a marker of chondrogenic differentiation.

All the primers used are listed in Table 1.

**Table 1:** List of primers used to evaluate the expression of genes related to trilineage differentiation genes by real-time PCR

Gene	Primer Sequence	Amplicon length (bp)
PPARγ	F: GCAGGAGCAGAGCAAAGAG R GAGGAGAGTTACTTGGTCGTTC	19 22
Adipophilin	F: GGGTAGAGTGGAAAAGGAGCAT R GATGTTGGACAGGAGGGTGTG	2 21
Runx2	F: AATGATGGTGTTGACGCTGA R TTGATACGTGTGGGATGTGG	20 20
OPN	F: TGGGAGGGCTTGGTTGTC R TTCCTTGGTCGGCGTTTG	18 18
Col1a1	F: CAATGCTGCCCTTTCTGCTCCTTT R: ATTGCCTTTGATTGCTGGGCAGAC	24 24
Sox9	F: AAAGGCTACGACTGGACG R CGGCTGGTACTTGTAATCC	18 19

### Karyotype analysis

Briefly, hUC-MSCs cultures were arrested in their exponential phase of growth by treating the cells with KaryoMax colcemid (Gibco) treatment for 2 h at 37 °C. The cells were harvested by trypsinization and subjected to hypotonic treatment with 0.075 M KCl followed by incubation at 37 °C for 15 min. Thereafter, a cell fixation step was performed using a methanol: glacial acetic acid (3:1) solution. For slide preparation, the cell suspension was added dropwise to cold slides followed by drying at 40–42 °C for 1–2 min. The slides were subjected to methanol and trypsin treatment, and before staining, they were washed with cold water. The slides were stained with modified Giemsa staining solution (Beyotime, China) for an optimized period. The cells were then observed under a light microscope (Martins et al.^2014^).

### Statistical analysis

The statistical analysis was performed using one-way ANOVA with Tukey’s post hoc multiple comparison test to compare the data between the fresh and cryopreserved hUC-MSC groups. The multilineage differentiation gene expression data obtained before and after 360 days of cryopreservation were compared using a paired t-test. All data collected were presented as a mean ± standard deviation (SD). Data analysis was performed using Graphpad Prism 7.0. All experiments were performed in triplicate and repeated three times. Values of *p* < 0.05 were considered statistically significant.

## Results

### Characterization of hUC-MSCs

**Fig. 1:**
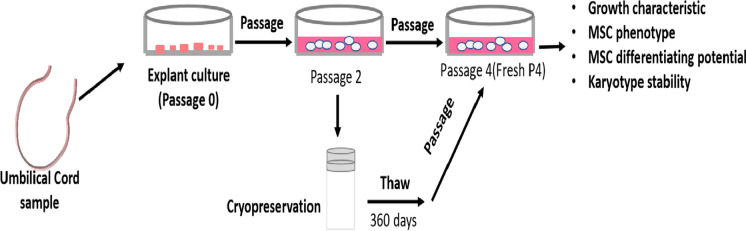
An overview of the study design

**Fig. 2:**
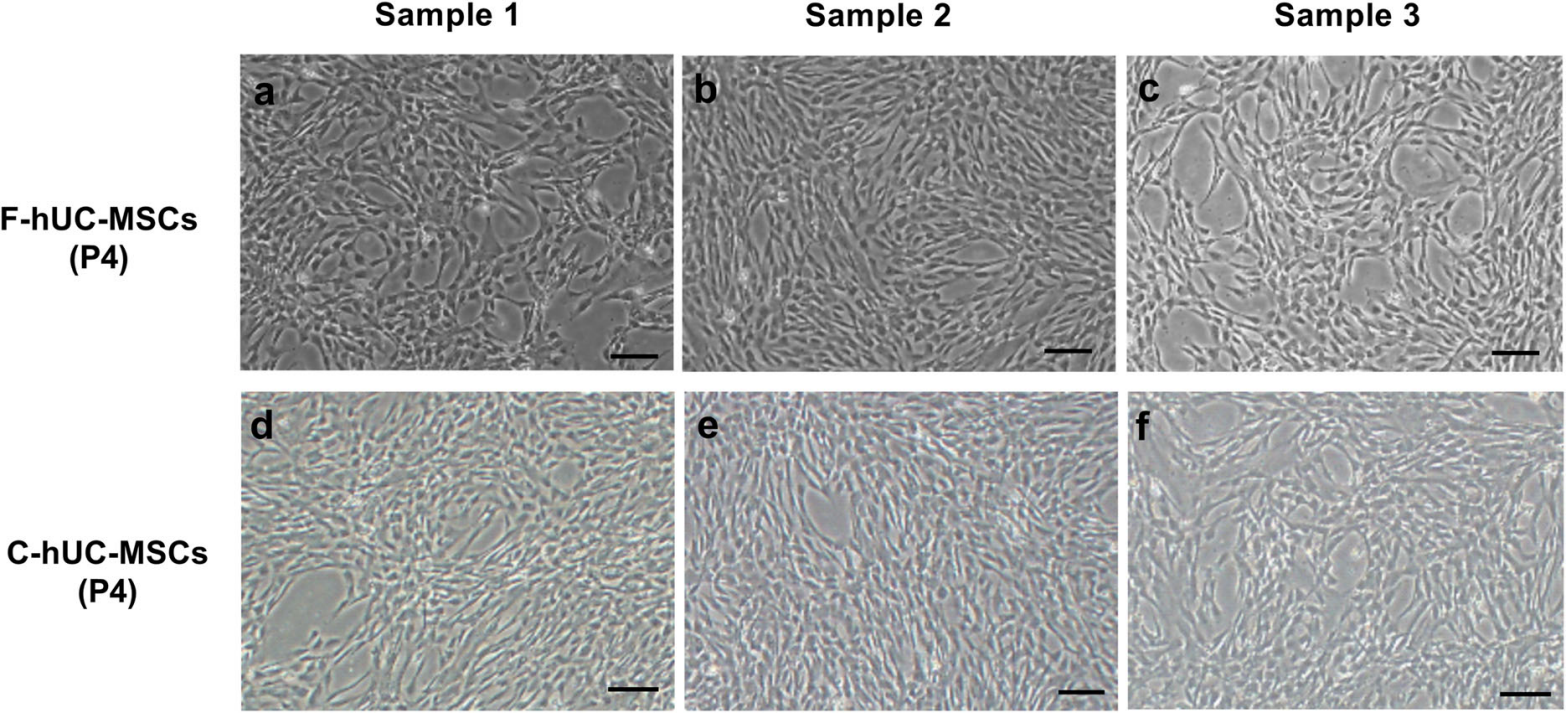
The biological characteristics of hUC-MSCs. Morphology of fresh (**a, b, c**) and cryopreserved hUC-MSCs (**d, e, f**) at passage 4, with a typical fibroblast-like morphology. Scale bar = 200 µm

hUC-MSCs were obtained from umbilical cord tissues from 3 different donors, and the ages of these mothers, were all over 30 years. The protocol for evaluating the basic characteristics of the cryopreserved hUC-MSCs is shown in Fig. 1. The original hUC-MSCs showed a spindle shape, had a typical fibroblast-like morphology, and exhibited colony formation abilities (Fig. 2a–c). The cell counts at passage 1 of all three samples are shown in Table 2, and we harvested approximately 170–280 million cells from approximately 15 to 20 cm of processed umbilical cord. All the primary cells were free of microbial, mycoplasma and endotoxin contamination (Table 3). By comparison with the STR database, it was found that these hUC-MSCs were distinct from one other (Table 4).

**Table 2:** Details of all the three fresh samples

	Mother age	Sex of baby	Gestation (weeks)	Cell yields at P1 (× 10^7^)
Sample 1	36	Male	37	21
Sample 2	33	Male	40	17
Sample 3	32	Male	39	28

**Table 3:** Initial harvest biosafety quality controls for all three fresh samples

	Eligibility criteria	Sample 1		Sample 2		Sample 3	
		P0	P4	P0	P4	P0	P4
Sterility	Sterile	Negative	Negative	Negative	Negative	Negative	Negative
Mycoplasma	Absence of mycoplasma	Absence	Absence	Absence	Absence	Absence	Absence
Endotoxin	< 0.5EU/ml	< 0.05EU/ml	< 0.05EU/ml	< 0.05EU/ml	< 0.05EU/ml	< 0.05EU/ml	< 0.05EU/ml

**Table 4:** Genotyping results of STR for all three fresh samples

	Sample 1	Sample 2	Sample 3
Locus	Allele	Allele	Allele
AMEL	X,Y	X,Y	X,Y
CSF1P0	11	12,13	9,10
D135317	8,10	13	9,10
D165539	11	12	12
D5S818	10,13	12	9,12
D7S820	11	8,9	9,13
TH01	9	9	9,9
TPOX	8,11	8,9	11
vWA	16,19	15,18	17,18

### Effect of cryopreservation on the phenotypic characterization of hUC-MSCs

**Fig. 3:**
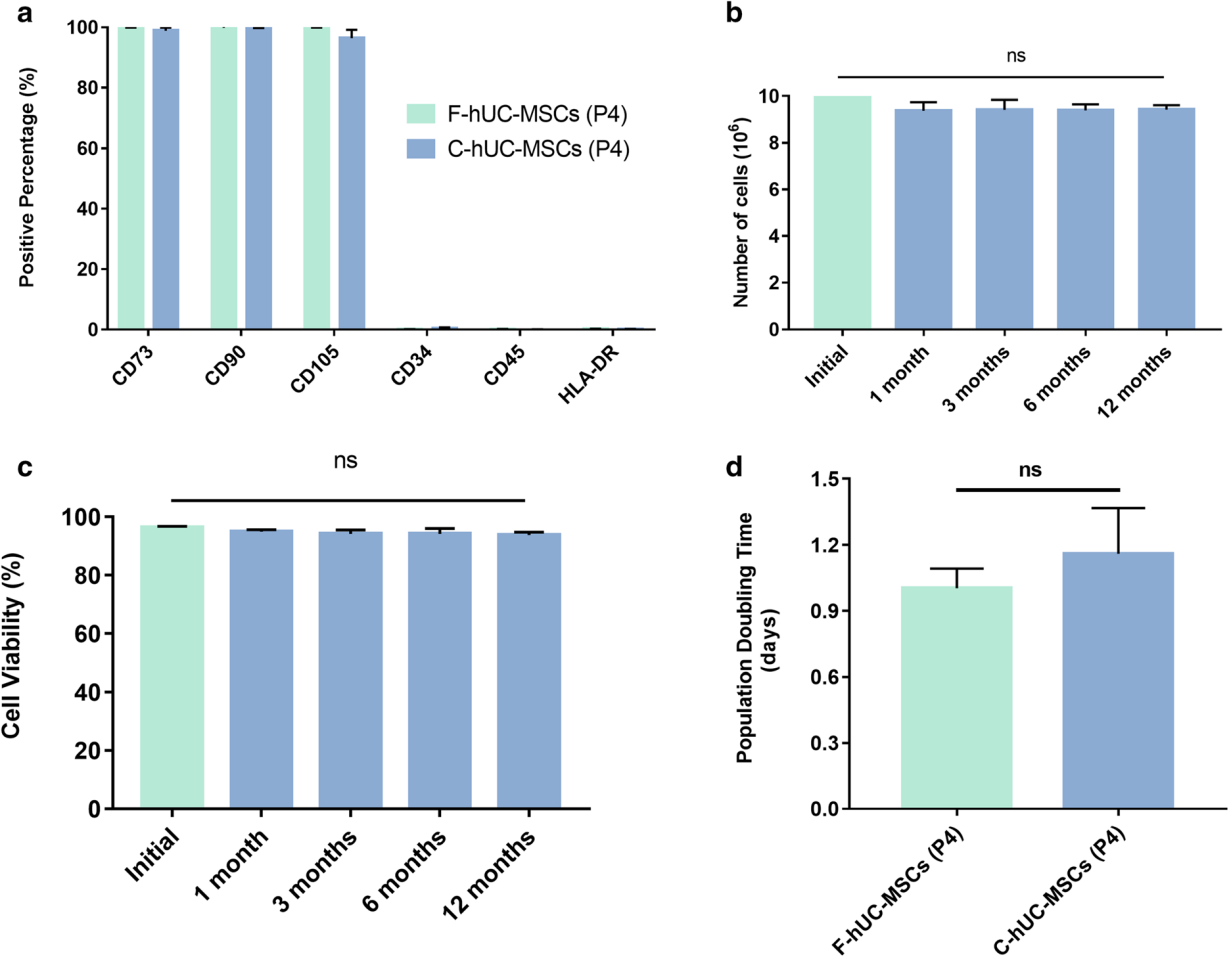
Comparison of the cell viability of fresh hUC-MSCs and cryopreserved hUC-MSCs.There were no significant differences in the expression of positive and negative surface marker of mesenchymal stem cell by fresh and cryopreserved hUC-MSCs at P4 (**a**). The total number of cells was no significant between the two groups (means ± sd) (**b**). Cell viability analysis revealed similar cell viability in fresh hUC-MSCs and hUC-MSCs cryopreserved for 12 months (**c**). Population doubling time (means ± sd) was estimated across passages for hUC-MSCs (**d,** n = 3 donors, experiments repeated three times in duplicate). Results expressed as mean ± SD. Comparision between groups performed using one-way ANOVA with Tukey’s post hoc multiple comparison tests. F-hUC-MSCs (P4): fresh human umbilical cord mesenchymal stem cells at passage 4; C-hUC-MSCs (P4): human umbilical cord mesenchymal stem cells at passage 4 after cryopreservation for one year

To investigate the effect of 12 months of cryopreservation on the hUC-MSCs phenotype, cryopreserved hUC-MSCs were thawed and cultured for two generations; the cell morphology studies revealed that fresh and cryopreserved hUC-MSCs at passage 4 presented adherent and normal spindle shapes, typical of mesenchymal stem cells (Fig. 2d–f). Flow cytometry analysis showed that fresh and cryopreserved hUC-MSCs were positive for CD73, CD90, and CD105 expression but negative for CD34, CD45, and HLA-DR expression (Fig. 3a). These results indicated that compared with fresh hUC-MSCs, cryopreserved hUC-MSCs retained their fibroblast-like shape and exhibited a similar cell surface marker expression pattern after 12 months of cryopreservation. Taken together, our findings suggested that the phenotypes of hUC-MSCs were not affected by long-term cryopreservation with serum-free cryopreservation medium.

### Effects of cryopreservation on hUC-MSCs count, viability, and proliferation

In addition to phenotype maintenance, cell survival and proliferation are critical factors for the applications of hUC-MSCs in the regenerative medicine field. After harvested, 1 × 10^7^ hUC-MSCs at passage 2, with an average viability of 96.34%, were stored in liquid nitrogen. The cell count and viability were tested after 1, 3, 6 and 12 months of cryopreservation. After thawing, the total cell counts at each time point were (0.938 ± 0.37) × 10^7^, (0.942 ± 0.43) × 10^7^, (0.939 ± 0.26) × 10^7^, and (0.943 ± 0.19) × 10^7^, respectively (Fig. 3b). The cell viabilities were 96.34% ± 0.43, 94.95% ± 0.71, 94.19% ± 1.35, 94.17% ± 1.88, and 93.81% ± 1.02, respectively (Fig. 3c). We also assessed cellular proliferation and found that cryopreserved and fresh hUC-MSCs displayed similar cell doubling times when cultured from passage 2 to passage 4 (Fig. 3d), suggesting that cryopreserved hUC-MSCs have a proliferation rate that is similar to that of fresh hUC-MSCs. The comparison between freshly passaged and cryopreserved/thawed hUC-MSCs revealed no significant difference in terms of cell viability and proliferation potential.

### Effects of cryopreservation on hUC-MSCs differentiation potential

**Fig. 4:**
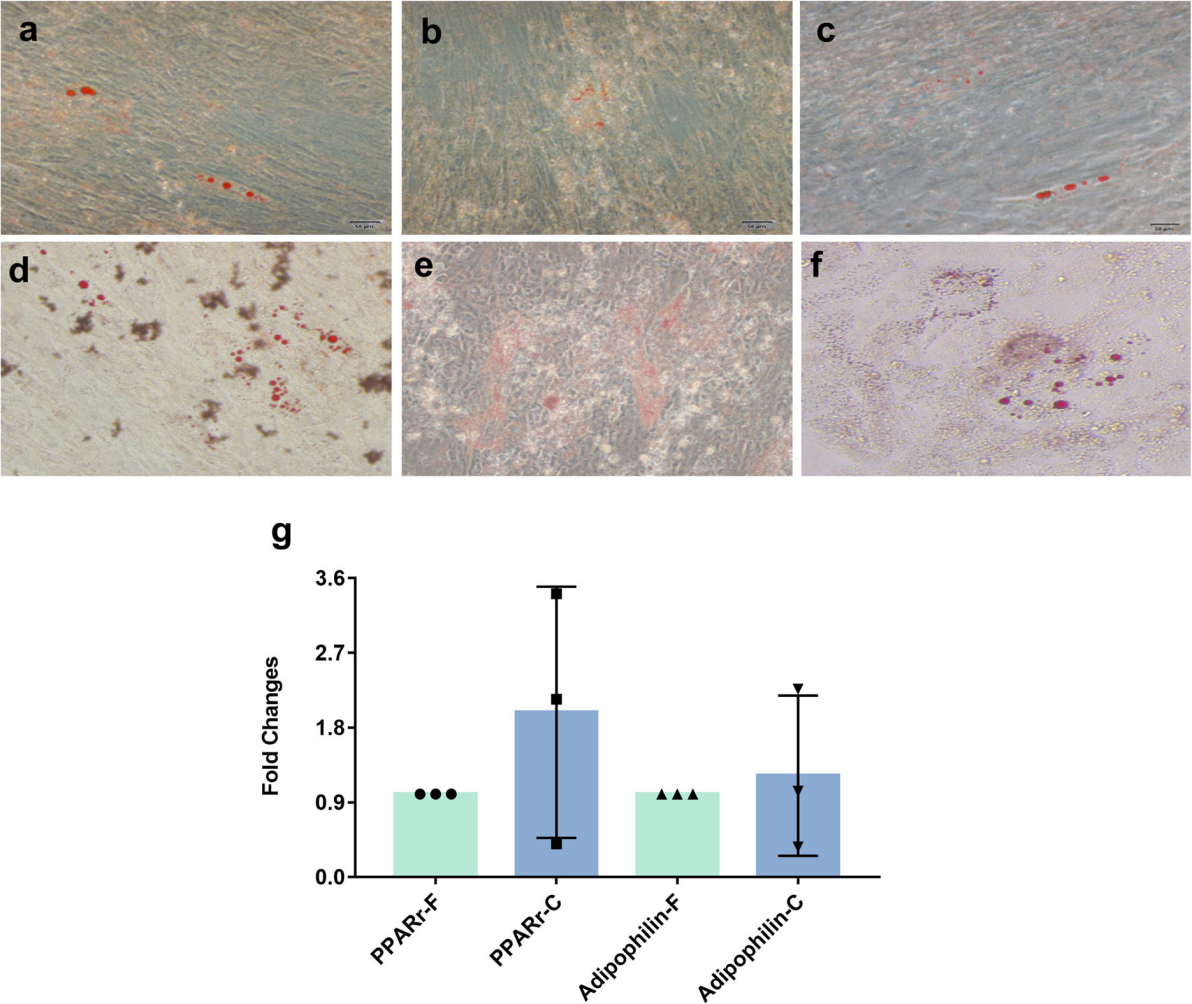
Adipogenic differentiation analysis of fresh hUC-MSCs and cryopreserved hUC-MSCs. Adipogenic differentiation analysis by Oil Red O staining of fresh hUC-MSCs (**a,b,c**) and cryopreserved hUC-MSCs (**d,e,f**). Similar adipogenic gene (PPARγ and Adipophilin) expression levels in C-hUC-MSCs compared to F-hUC-MSCs (**g**). Results are expressed as fold change relative to fresh group. Comparision between groups was performed by paired t-test

**Fig. 5:**
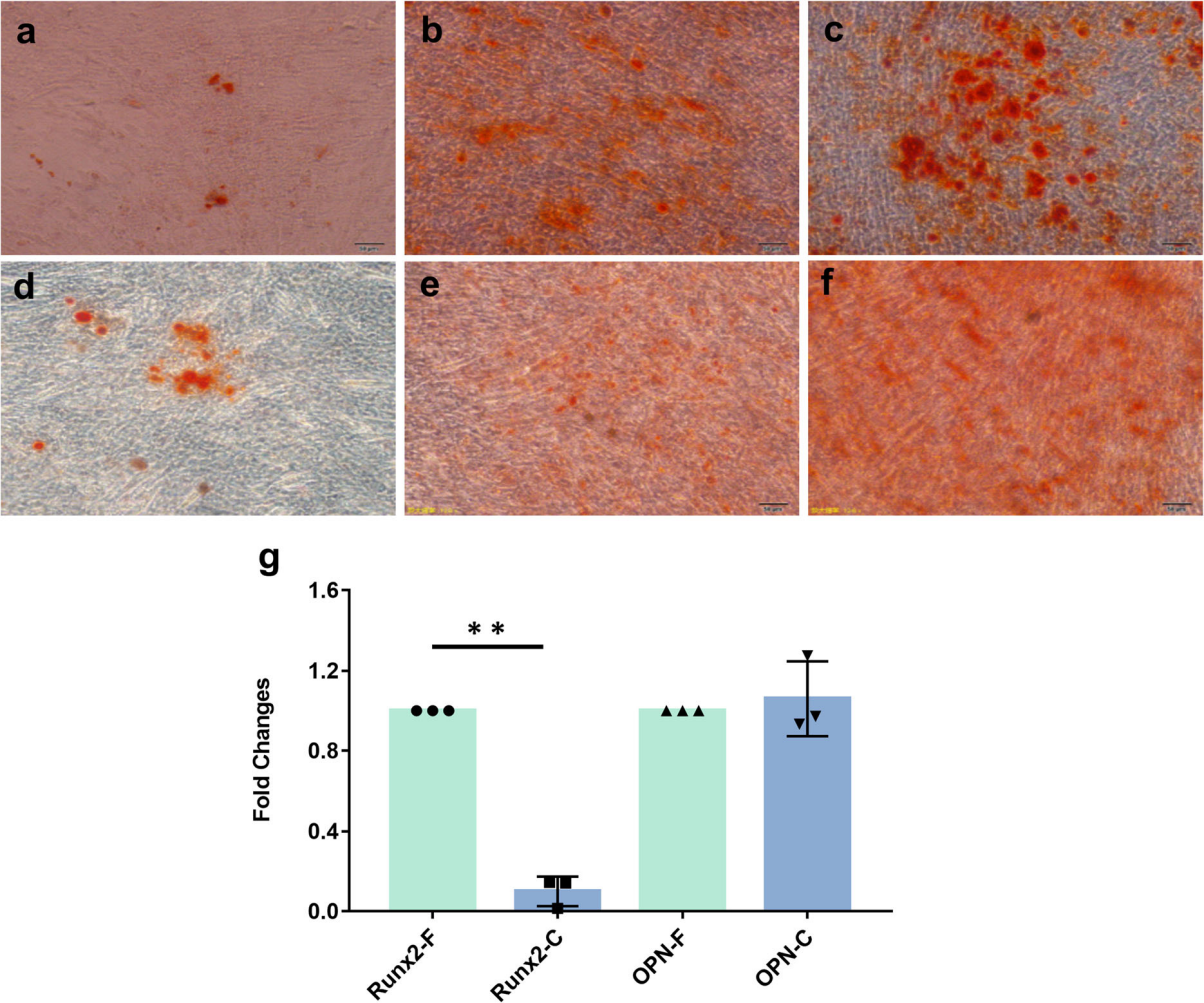
Osteogenesis differentiation analysis of fresh hUC-MSCs and cryopreserved hUC-MSCs. Osteogenesis differentiation analysis by Alizarin Red staining of fresh hUC-MSCs (**a,b,c**) and cryopreserved hUC-MSCs (**d,e,f**). C-hUC-MSCs expressed decreased levels of osteogenic marker Runx2 compared to F-hUC-MSCs. **P < 0.01 relative to fresh hUC-MSCs after 21 days of osteogenic induction (**g**). Results are expressed as fold change relative to fresh group. Comparision between groups was performed by paired t-test. **P < 0.01 is considered statistically significant

**Fig. 6:**
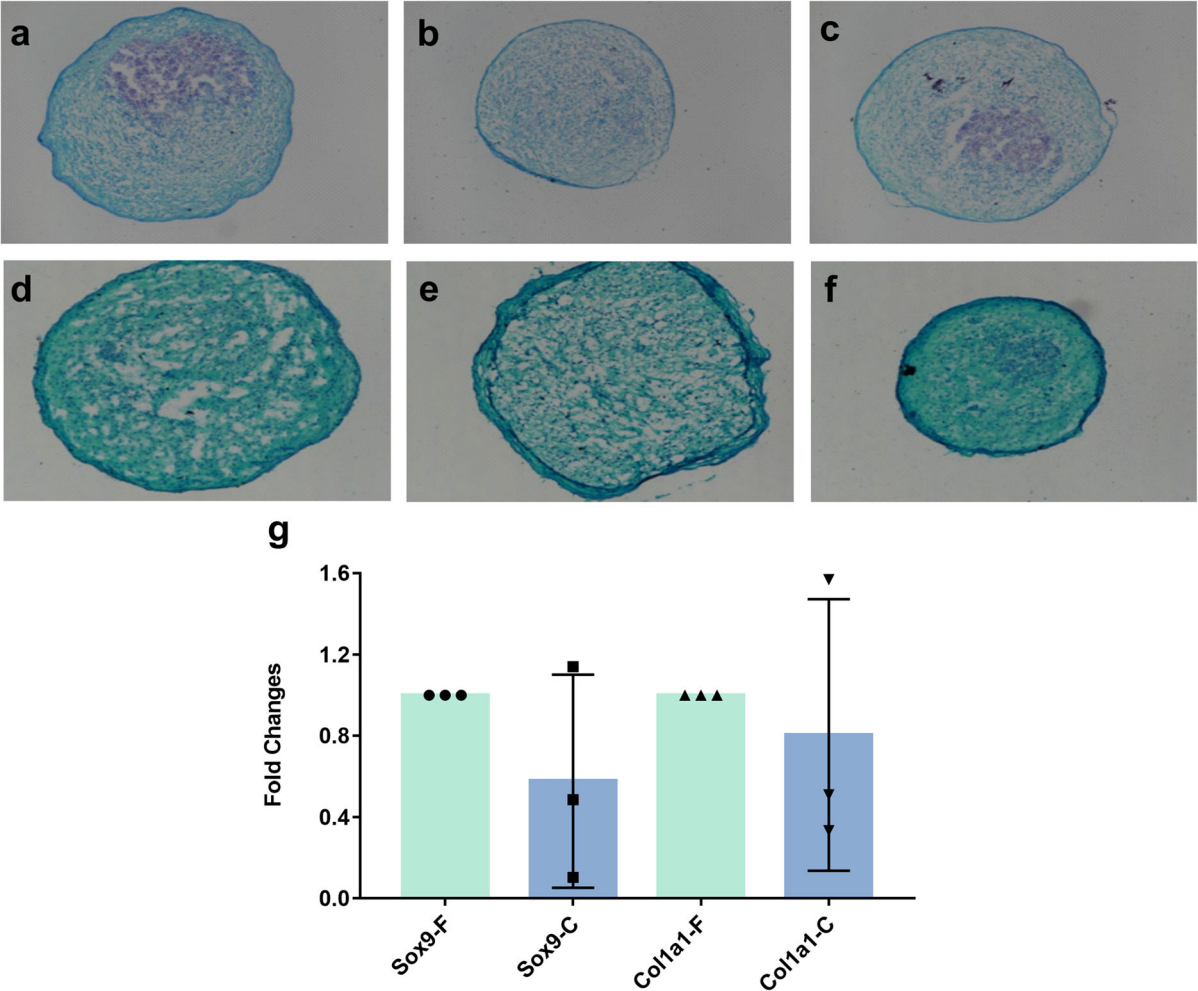
Chondrogenesis differentiation analysis of fresh hUC-MSCs and cryopreserved hUC-MSCs. Chondrogenesis differentiation analysis by Alcian Blue staining of fresh hUC-MSCs (**a, b, c**) and cryopreserved hUC-MSCs (**d, e, f**). C-hUC-MSCs expressed decreased levels of chondrogenic markers (Sox9 and Col1a1), but there was no significant difference (**g**). Results are expressed as fold change relative to fresh group. Comparision between groups was performed by paired t-test

Furthermore, the multipotency of hUC-MSCs was evaluated by staining for differentiation markers and analyzing the expression of differentiation marker genes. After adipogenic differentiation, both fresh and cryopreserved/thawed hUC-MSCs exhibited lipid droplet formation and positive staining by Oil Red O, indicating that cryopreserved hUC-MSCs maintained their adipogenic potential and could be differentiated by simulation with adipogenic medium for 21 days (Fig. 4a–f). Moreover, under osteogenic conditions, fresh and freeze-thawed hUC-MSCs exhibited positive Alizarin Red staining (Fig. 5a–f). For the chondrogenic potential of cryopreserved/thawed hUC-MSCs was determined by histological section staining with Alcian Blue. These pellets were positively stained with Alcian Blue with varying degrees of intensity (Fig. 6a–f).

To investigate the molecular changes in response to cryopreservation, the expression of specific differentiation-related genes in hUC-MSCs was measured by real-time PCR. We observed that after adipogenic induction, the adipogenic genes PPARγ and adiponectin were expressed at higher levels in the cryopreserved hUC-MSCs group than in the fresh hUC-MSCs group. However, due to large individual variation, the expression of these genes in the cells from donor 2 and donor 3 after cryopreservation was altered by twofold or more, while the expression of these genes in the cells from donor 1 was altered by less than twofold, compared to that in freshly differentiated; thus, the expression of adipogenic-related genes was not statistically significant (Fig. 4g). Regarding osteogenic induction, cryopreserved/thaw hUC-MSCs showed significantly decreased expression of Runx2 (gene expressed in the early stage of osteogenic differentiation), but the osteogenesis-related gene OPN (mature osteocyte marker) was not significantly different. The results confirmed that cryopreserved hUC-MSCs could maintain osteogenic differentiation potential, while cryopreserved hUC-MSCs showed a decreased capacity for osteogenic differentiation compared with fresh hUC-MSCs (Fig. 5g). Similarly, cryopreserved hUC-MSCs showed decreased expression of the chondrogenic markers Sox9 and Col1a1 after 21 days of chondrogenic induction (Fig. 6g). The differences did not reach statistical significance. The real-time PCR results were consistent with the staining results.

### Effects of cryopreservation on hUC-MSCs cytogenetics

**Fig. 7:**
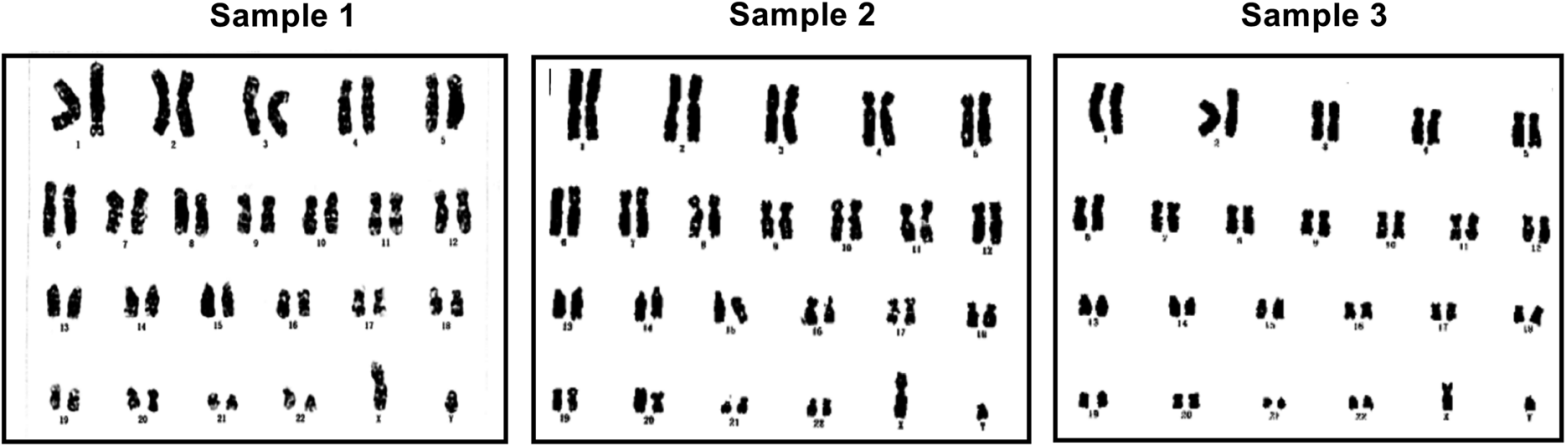
Karyotype of cryopreserved hUC-MSCs from three donors. The normal karyotype of hUC-MSCs was shown by G-banding after storage for one year (**a, b, c**)

Karyotype analysis was performed to test the chromosomal stability of hUC-MSCS after 12 months of cryopreservation. The results showed that no numerical or structural abnormalities were observed in the karyotype as shown in Fig. 7.

## Discussion

In the present study, we investigated the post-thaw recovery of hUC-MSCs that had been stored at -196°C for up to 12 months by using commercial serum-free freezing medium (CellBanker 2). We observed that the cryopreserved hUC-MSCs retained small spindle-like morphological characteristics, cell proliferation potential, specific cell surface marker expression, multilineage differentiation potential and chromosomal stability. These data suggested that the cryopreservation of hUC-MSCs met the criteria approved by the International Society for Cell Therapy, and the biological characteristics of hUC-MSCs were maintained after cryopreservation for 12 months in commercial serum-free freezing medium.

Previous studies have shown that cyropreserved hUC-MSCs maintain their cell morphology, surface cell marker expression and original proliferation ability after long-time cryopreservation in different cryoprotectant media (Fu et al. 2020; Shivakumar et al. 2015). These findings are basically consistent with our conclusion that hUC-MSCs that were cryopreserved for one year retained their biological properties. In addition, the cryopreservation of mouse and human ADSCs was evaluated using commercial cryopreservation solutions (CellBanker 2), and the results indicated that CellBanker 2 could yield a better survival rate after freezing and thawing (Miyagi-Shiohira et al.^2015^; Oishi et al.^2008^). Indeed, our study found 93.81% viability for the frozen and thawed hUC-MSCs at passage 2, which to the best of our knowledge, is superior to that observed in other published reports. These factors may benefit both cell transplantation and longer cryopreservation. Collectively, these data support the reliability and consistency of cryopreserved hUC-MSCs.

Furthermore, the regulation of the directional differentiation of MSCs is the core issue for enhancing MSC-mediated tissue regeneration. Previous studies have demonstrated conflicting results regarding the relationship between adipogenesis and osteogenesis (James 2013; Yuan et al. 2016). The results of our study also verify this idea. In this study, we used a commercial serum-free freezing medium (CellBanker 2) with programmed slow freezing to preserve the cells. We observed that cryopreserved/thawed hUC-MSCs retained their multipotency and could be induced to differentiate into the adipogenic, osteogenic and chondrogenic lineages. The differentiation potential, as evaluated by histochemical staining and gene expression analyses, showed the upregulated expression of specific adipogenic-related genes in the cryopreservd/thawed hUC-MSCs after one year compared with that in the fresh hUC-MSCs. However, the osteogenic potential of cryopreserved hUC-MSCs was impaired in the cryopreserved group, which was shown by the reduction in Runx2 gene expression compared with that of the fresh hUC-MSCs. Interestingly, no significant differences were observed in terms of the mean expression of OPN between the cryopreserved hUC-MSCs and fresh hUC-MSCs from the same donors. Runx2 is an early osteogenic marker obeserved at the early stage of differentiation, which OPN expression is observed at the middle/late stage of differentiation (Xu et al. 2015)0. A variety of bone growth factors may participate in the regulation of bone differentiation and bone metabolism. For instance, BMP2, an osteogenic factor, plays an important role in regulating the expression of genes related to the mineralization process during osteoblast differentiation (Wagner et al. 2010). Thus, we presume that other osteogenic factors might participate in the process of osteogenic differentiation and promote the expression of OPN. A similar decrease in the osteogenic potential of frozen-thawed ASCs was also previously reported by James et al. (James et al., 2010)0.0 In addition, the expression of chondrogenic markers Sox9 and Col1a1 was also decreased in cryopreserved hUC-MSCs compared with fresh hUC-MSCs. Fu et al. reported that the expression of complement C3, a component of the innate immune system, was increased by 3.729 fold in cryopreserved hUC-MSCs compared with fresh hUC-MSCs, as shown by mass spectrometry-based proteome profile analysis (Hoogduijn et al.^2016^). A previous study showed that C3 could enhance adipogenic differentiation (Rouaud et al. 2017)0.0 Therefore, we hypothesized that this might cause the adipogenic differentiation ability of cryopreserved hUC-MSCs to increase.

The effect of cryopreservation on the therapeutic properties of MSCs has become highly controversial. Some recent reports suggest that cryopreserved and thawed MSCs may have impaired functional properties as compared to freshly harvested MSCs (Shaik et al. 2018; Chinnadurai et al. 2014). In contrast, some recent conflicting studies have shown that the functionality and cell characteristics of cryopreserved MSCs are comparable to those of fresh MSCs (Oja et al.^2019^). These conflicting results warrant further studies to elucidate the impact of a cryopreservation step in the manufacture of clinical-grade MSCs. In this study, we used xeno-free hPL supplemented medium to expansion of hUC-MSCs and cryopreserve hUC-MSCs by commercial serum-free freezing medium (CellBanker 2), which contain DMSO, glucose, a prescribed high polymer, and pH adjustors, allowing the cryopreservation of cells for long time. These results demonstrated that hUC-MSCs retained strong proliferative activities after one year of cryopreservation, which is a critical factor for cell transplantation-based therapies. In addition, we validated the stability of the cellular karyotype, while previous studies have paid little attention to this stem cell property. Our findings provide evidence for the application of cryopreserved hUC-MSCs in the regenerative medicine field.

hUC-MSC-based therapy is attractive due to the differentiation potential, immunomodulatory properties, and paracrine effects of hUC-MSCs. The limitations of our study lie in the incomplete understanding of the immunosuppressive and paracrine effects of hUC-MSCs. Thus, future work will pursue this aspect of analysis.

## Conclusion

In this study, we evaluated and compared the biological characteristics of hUC-MSCs after cryopreservation for 12 months to those of fresh hUC-MSCs. Plastic-adherent hUC-MSCs can be expanded in hPL-supplemented culture medium. The morphology, surface markers expression, cell viability and proliferative potential of hUC-MSCs were tested after cryopreservation with the commercial serum-free cryoprotective agent CellBanker 2 and subculturing, and these characteristics were compared with those of fresh hUC-MSCs, the results showed no obvious differences between the two groups. Cryopreservation can maintain the multilineage differentiation capacities of hUC-MSCs and fulfill ISCT minimal criteria. However, compared to noncryopreserved hUC-MSCs from the same donor, cryopreserved hUC-MSCs demonstrated reduced osteogenic and chondrogenic potential and enhanced adipogenic potential. These results will be helpful for understanding the biological processes that occur during stem cell cryopreservation and will promote the improvement of cryopreservation facilities and techniques to meet the requirements of clinical application. The study will also provide indispensable new references for the further clinical application of hUC-MSCs.

## Declarations

### Data availability statement

All data, models, and code generated or used during the study appear in the submitted article.
